# Proton pump inhibitor therapy usage and associated hospitalization rates and critical care outcomes of COVID-19 patients

**DOI:** 10.1038/s41598-022-11680-0

**Published:** 2022-05-09

**Authors:** Brittney Shupp, Sagar V. Mehta, Subin Chirayath, Nishit Patel, Mina Aiad, Jared Sapin, Jill Stoltzfus, Yecheskel Schneider

**Affiliations:** 1 Internal Medicine Resident, St. Luke’s University Health Network, Bethlehem, PA USA; 2 Department of Gastroenterology, St. Luke’s University Health Network, Bethlehem, PA USA; 3 Internal Medicine Resident, St. Luke’s University Health Network, Easton, PA USA; 4 Lewis Katz School of Medicine at Temple University – St. Luke’s Campus, Bethlehem, PA USA; 5 St. Luke’s University Health Network, Bethlehem, PA USA; 6 Division of Internal Medicine, St. Luke’s University Health Network, 801 Ostrum St., Suite 201, Bethlehem, 18015 PA USA

**Keywords:** Viral infection, Risk factors

## Abstract

Proton Pump Inhibitors (PPI) are one of the most prescribed medications in the United States. However, PPIs have been shown to increase the risk of enteric infections. Our study aims to evaluate the correlation between PPI and COVID-19 severity. We performed a retrospective cohort study on patients who tested positive for SARS-CoV-2 from March to August 2020. Patients were categorized based on PPI user status. Primary outcomes included need for hospital or ICU admission and 30-day mortality. Secondary outcomes looked to determine the severity of COVID-19 infection and effect of comorbid conditions. 2,594 patients were reviewed. The primary outcomes of our study found that neither active nor past PPI use was associated with increased hospital admission or 30-day mortality following completion of multivariate analysis. Additionally, there was no association between COVID-19 infection and the strength of PPI dosing (low, standard, high). However, the following covariates were independently and significantly associated with increased admission: age, male gender, diabetes, COPD, composite cardiovascular disease, kidney disease, and obesity. The following covariates were associated with increased mortality: age, male gender, COPD, and kidney disease. In conclusion, the high risk features and comorbidities of PPI users were found to have a stronger correlation to severe COVID-19 infection and poor outcomes as opposed to the use of PPI therapy.

## Introduction

SARS-CoV-2 or Coronavirus Disease 2019 (COVID-19) is a viral disease that has surmounted into a global pandemic immensely impacting healthcare in the United States (US) and around the world. As of March 2022, there are over 446 million worldwide cases of recorded COVID-19 infections with over 80 million in the US alone and millions of high-risk individuals who remain unvaccinated^1^. The clinical manifestations of COVID-19 vary widely; however, those with severe COVID-19 illness typically have significant respiratory compromise^2–5^. Several risk factors for both susceptibility of infection and clinical outcomes have been proposed, including age greater than 65, diabetes, coronary artery disease and chronic obstructive pulmonary disorder (COPD) placing individuals at increased risk^6,7^. Additionally, proton pump inhibitor (PPI) use has been identified as a possible risk factor for increased severity for COVID-19 infection, yet this association has not been extensively studied.

PPIs are one of the most common classes of medications prescribed in the US^8^. Their use however has been associated with increased risk of infections including pneumonia, Clostridium difficle and spontaneous bacterial peritonitis^9–13^. It is postulated that these infections may occur due to a decrease in gastric acid leading to a disruption of gut flora^9^. Studies evaluating the relationship between the severity of COVID-19 infection and PPI therapy are emerging, but the relationship is not well established. Therefore, our study aims to determine the association between the severity of COVID-19 infection and trends in PPI use.

## Methods

### Study design

We performed a retrospective cohort study within St. Luke’s University Health Network (SLUHN), a 10-hospital network located in Eastern Pennsylvania. This study was approved by the SLUHN Institutional Review Board (IRB) and was performed in accordance with institutional guidelines and regulations. Charts of patients who underwent COVID-19 testing where reviewed and those who tested positive for SARS-CoV-2 through nasopharyngeal swab specimens and SARS-CoV-2 reverse transcriptase polymerase chain reaction (RT-PCR) testing from March 2020 to August 2020 met inclusion criteria. Patients who had repeated testing completed were only counted for once. Those who only had positive serological antibody testing, and not a positive RT-PCR, were excluded from the study. Charts were reviewed to determine patient’s history of PPI use (including esomeprazole, omeprazole, pantoprazole, lansoprazole, and rabeprazole).

### Data collection

Data was collected through utilization and review of the electronic health record (EHR) system, and all variables were recorded in one data collection form. Information regarding PPI use was collected including type, current status (active use, past use, or no use; active users had been prescribed and taking PPIs within the last 30 days prior to admission while past users included those who had a history of usage within the last 31 to 365), strength (grouped as low, standard, and high; based off clinical guidelines published by the National Institute for Health and Care Excellence)^14^. Additional variables included critical care outcomes including need for supplemental oxygen, ICU admission, mechanical ventilation, and 28-day mortality were also collected.

### Data analysis

Using SPSS version 27 to analyze our data (Armonk, NY: IBM Corp), we first compared patient demographic and clinical variables between our three patient groups (patients with active PPI use, patients with past PPI use, and patients with no PPI use). Next, we constructed direct multivariable logistic regression models to determine the independent effect of PPI usage on hospital admission within two weeks of COVID testing and 30-day mortality after adjusting for relevant patient covariates. Although we originally planned to model hospital discharge disposition as an additional outcome, there were insufficient subgroup samples for several categories, so we reported only descriptive information.

Prior to regression modeling, we conducted separate bivariate analyses (one-way analysis of variance for normally distributed continuous variables and chi square tests for categorical variables) to determine which covariates were best suited to multivariate modeling for each of our three outcomes at *p* < 0.20. In addition to PPI usage, our potential covariates included age; gender; race (white versus non-white/other/did not answer due to small subgroup sizes for nonwhite racial groups); diabetes; chronic obstructive pulmonary disease (COPD); composite cardiovascular disease (heart failure, cardiomyopathy, and/or coronary artery disease); kidney disease; cancer; and obesity [defined as body mass index (BMI) > 30]. We were unable to include sickle cell anemia or organ transplantation due to limited samples sizes within each PPI group.

We also assessed for linearity in the logit for age and BMI as continuous covariates; both had acceptable values for all models. Based on examination of the normalized residuals, Cook’s D, and leverage statistics, the admission model had 124/2,593 outliers (4.8%); the SNF residency model had 67/2,592 outliers (2.6%); and the 30-day mortality model had 61/1,757 outliers (3.5%); there were no influential data points for any of the models. Given these relatively small values, we retained all patients in our regression analyses.

To ascertain model goodness of fit, we reported the omnibus chi square statistic and the HosmerLemeshow goodness-of-fit statistic. For each covariate, we present adjusted odds ratios (AOR), and 95% confidence intervals (CIs), with *p* < 0.05 denoting statistical significance for covariates in the final models.

We further evaluated hospital-admitted patients based on their PPI usage by conducting separate chi square and Kruskal Wallis tests for the following categorical and skewed continuous outcomes, respectively: 1) COVID versus non-COVID reason for admission; 2) oxygen usage; 3) ICU admission; 4) hospital length of stay; and 5) distribution of comorbidities. Finally, we evaluated only active PPI users based on their PPI dosages by conducting separate chi square and Kruskal Wallis tests for the following outcomes: 1) hospital admission within two weeks of COVID testing; 2) oxygen usage; 3) ICU admission; 4) hospital length of stay; and 5) 30-day mortality. For 7 these analyses, *p* < 0.05 denotes statistical significance, with no adjustment for multiple comparisons.

### Ethical approval and consent to participate

Ethics approval was obtained from the Institutional Review Board (IRB) before starting the study. No consent to participate was taken or needed (with approval from IRB) as study was retrospective in nature and based of the review of patient charts.

## Results

A total of 2,594 patient charts were reviewed and included in the study sample. 1,312 subjects were female (50.5%) and 1,499 (57.7%) were white. The mean age was 52.6 years and the mean BMI was 30.7. 2,048 patients (78.9%) had no past or present history of PPI use. 448 individuals (17.3%) were active PPI users and 98 individuals (3.8%) had a history of PPI use. Key demographic and clinical characteristics of each individual group are listed in Table 1. Those in the active or prior PPI use group were associated with significantly higher rates of concurrent diabetes, COPD, compositive cardiovascular disease (cardiomyopathy, congestive heart failure, and coronary artery disease), kidney disease, cancer, and obesity.

**Table 1:** Patient demographic and clinical variables*.

	Active PPI use (n = 448)	Past PPI use (n = 98)	No PPI use (n = 2,048)	*p*-value*****
Age, years (mean ± SD)**	65.0 ± 17.2	62.2 ± 18.6	49.5 ± 19.8	< .0001
Gender (n,%)	255 female (56.9%) 193 male (43.1%)	64 female (65.3%) 34 male (34.7%)	990 female (48.3%) 1,058 male (51.7%)	< .0001
Race (n,%)	309 white (69%) 139 non-white/other/did not answer (31%)	57 white (58.2%) 41 non-white/other/did not answer (41.8%)	1,129 white (55.1%) 919 non-white/other/did not answer (44.9%)	< .0001
BMI, kg/m2 (mean ± SD)**	30.9 ± 9.2	30.3 ± 6.6	30.7 ± 7.6	.84
Diabetes (n,%)	200 (44.6%)	46 (46.9%)	479 (23.4%)	< .0001
COPD* (n,%)	111 (24.8%)	21 (21.4%)	134 (6.5%)	< .0001
Cardiovascular disease*** (n,%)	213 (47.5%)	48 (49%)	297 (14.5%)	< .0001
Kidney disease (n,%)	153 (34.2%)	33 (33.7%)	222 (10.8%)	< .0001
Cancer (n,%)	93 (20.8%)	18 (18.4%)	136 (6.6%)	< .0001
Obesity**** (n,%)	249 (55.6%)	50 (51%)	681 (33.3%)	< .0001
Organ transplant (n,%)	1 (0.2%)	0	7 (0.3%)	.79
Sickle cell anemia (n,%)	7 (1.6%)	3 (3.1%)	13 (0.6%)	.01

*Denominators differ for some variables due to missing data.

**SD Standard deviation, *COPD* Chronic obstructive pulmonary disease.

***Cardiovascular disease is a composite of cardiomyopathy, congestive heart failure, and coronary artery disease.

****Defined as BMI > 30 kg/m2.

*****Based on separate one-way analysis of variance or chi square tests, as appropriate.

Bivariate comparisons were completed looking at hospital admission within two weeks of COVID testing as well as overall 30-day mortality and are listed in Table 2. Of 1,040 total admissions, 286 (27.5%) were active PPI users, 54 (5.2%) past PPI users, and 700 (67.3%) non-PPI users. Bivariate comparisons identified the following 10 covariates for inclusion in the multivariable regression model (*p* < 0.20): PPI usage, age, gender, race, diabetes, composite cardiovascular disease, COPD, kidney disease, cancer, and obesity. With regards to 30-day mortality, 186 patients died in total: 56 (30.1%) active PPI users, 11 (5.9%) past PPI users, and 119 (64%) non-PPI users. Bivariate comparisons identified the following 10 covariates for inclusion in the multivariable regression model (*p* < 0.20): PPI usage, age, BMI, gender, race, diabetes, composite cardiovascular disease, COPD, kidney disease, and cancer.

**Table 2:** Unadjusted comparisons for hospital admission within two weeks of COVID testing and for overall 30-day mortality *.

	Admitted (n = 1,040)	Not Admitted (n = 1,553)	*P*- value*****	Alive (n = 2,408)	Deceased (n = 186)	*p*-value*****
PPI usage** (n,%)	Active use: 286 (27.5%) Past use: 54 (5.2%) No use: 700 (67.3%)	Active use: 162 (10.4%) Past Use: 44 (2.8%) No use: 1,347 (86.7%)	< .0001	Active use: 392 (16.3%) Past use: 87 (3.6%) No use: 1,929 (80.1%)	Active use: 56 (30.1%) Past Use: 11 (5.9%) No use: 119 (64%)	< .0001
Age, years (mean ± SD)**	66.5 ± 17.2	43.4 ± 16.5	< .0001	50.7 ± 19.4	77.7 ± 12.8	< .0001
Gender (n,%)	488 female (46.9%) 552 male (53.1%)	820 female (52.8%) 733 male (47.2%)	.003	1,229 female (51%) 1,179 male (49%)	80 female (43%) 106 male (57%)	.04
Race (n,%)	677 white (65.1%) 363 non-white/other/did not answer (34.9%)	817 white (52.6%) 736 non-white/other/did not answer (47.4%)	< .0001	1,352 white (56.1%) 1,056 non-white/other/did not answer (43.9%)	143 white (76.9%) 43 non-white/other/did not answer (23.1%)	< .0001
BMI, kg/m2 (mean ± SD)**	30.5 ± 8.4	30.9 ± 7.3	.28	31.0 ± 8.1	28.3 ± 5.9	< .0001
Diabetes (n,%)	498 (47.9%)	277 (14.6%)	< .0001	630 (26.2%)	95 (51.1%)	< .0001
COPD** (n,%)	227 (21.8%)	39 (2.5%)	< .0001	201 (8.3%)	65 (34.9%)	< .0001
Cardiovascular disease*** (n,%)	448 (43.1%)	109 (7%)	< .0001	445 (18.5%)	113 (60.8%)	< .0001
Kidney disease (n,%)	351 (33.8%)	56 (3.6%)	< .0001	308 (12.8%)	100 (53.8%)	< .0001
Cancer (n,%)	177 (17%)	70 (4.5%)	< .0001	190 (7.9%)	57 (30.6%)	< .0001
Obesity**** (n,%)	503 (48.4%)	477 (30.7%)	< .0001	905 (37.6%)	75 (40.3%)	.46

*Denominators differ for some variables due to missing data.

**PPI Proton pump inhibitor, *SD* Standard deviation, *COPD* Chronic obstructive pulmonary disease.

***Cardiovascular disease is a composite of cardiomyopathy, congestive heart failure, and coronary artery disease.

****Defined as BMI > 30 kg/m2.

*****Based on separate one-way analysis of variance or chi square tests, as appropriate.

Multivariable regression results are displayed in Table 3. In the regression model, PPI use was not associated with hospital admissions or 30-day mortality. The model found the following variables significant: age, male gender, diabetes, COPD, composite cardiovascular disease, kidney disease and obesity. As further presented in Table 3, the following covariates were significantly associated with increased mortality: age, male gender, COPD and kidney disease.

**Table 3:** Multivariable logistic regression for hospital admission within two weeks of COVID testing and 30-day mortality*.

	Hospital admission		30-day mortality	
	AOR (95% CI)**	*p*-value	AOR (95% CI)**	*p*-value
PPI Usage** *(ref* = *no use)*	1) Active use: 1.10 (.83–1.45) 2) Past use: .75 (.44–1.27)	.51 .28	1) Active use: .81 (.54–1.22) 2) Past use: .81 (.37–1.79)	1) .32 2) .61
Age	1.06 (1.05–1.07)	< .0001	1.08 (1.06–1.09)	< .0001
Gender *(ref* = *female)*	1.36 (1.11–1.67)	.004	1.93 (1.32–2.82)	.001
Race *(ref* = *white)*	1.09 (.88–1.34)	.45	.94 (.60–1.46)	.78
Diabetes	2.00 (1.59–2.52)	< .0001	1.28 (.87–1.86)	.21
COPD**	2.44 (1.62–3.67)	< .0001	2.03 (1.35–3.04)	.001
Cardiovascular Disease***	1.60 (1.18–2.16)	.002	.95 (.62–1.47)	.82
Kidney disease	2.45 (1.72–3.48)	< .0001	2.05 (1.38–3.06)	< .0001
Cancer	.92 (.63–1.33)	.66	1.18 (.76–1.82)	.47
Obesity/BMI****	1.81 (1.46–2.24)	< .0001	.99 (.97–1.02)	.68

*Omnibus chi-square *p*-value < .0001; Hosmer–Lemeshow goodness-of-fit p-value = .51 (For Hospital Admission within Two Weeks of COVID Testing) and p-value = .49 (For 30-Day Mortality).

**AOR Adjusted odds ratio, *CI* Confidence interval, *PPI* Proton pump inhibitor, *COPD* Chronic obstructive pulmonary disease, *BMI* Body Mass Index.

***Cardiovascular disease is a composite of cardiomyopathy, congestive heart failure, and coronary artery disease.

****Obesity utilized for a Hospital Admission within 2 Weeks of COVID Testing) and BMI utilized for 30-Day Morality; Defined as BMI > 30 kg/m2.

Secondary outcomes for admitted patients based on their PPI use was evaluated and are listed in Table 4. Compared to past and nonusers, active PPI users had slightly higher median hospital length of stayl (*p* = 0.02). Additionally, past PPI users had a higher percentage of ICU admissions (*p* = 0.03), while both active and past PPI users had higher percentages of diabetes, COPD, composite cardiovascular disease, kidney disease, cancer, and obesity (*p* < 0.006). For active and past PPI users (n = 546), pantoprazole was most frequently taken (316, 57.9%), followed by omeprazole (196, 35.9%); esomeprozole (19, 3.5%); lansoprazole (14, 2.6%); dexlansoprazole (5, 0.9%); and rabeprazole (2, 0.4%). Table 5 further presents secondary outcomes for active PPI users only based on dosage (low, standard, or high). None of the outcomes were significantly different.

**Table 4:** Hospital-admitted patient outcomes based on PPI Use* (n = 1,040).

	Active PPI use (n = 286)	Past PPI use (n = 54)	No PPI use (n = 700)	*p*-value*****
COVID-related admission (n,%)	228/286 (79.7%)	40/53 (75.5%)	583/695 (83.9%)	.11
Length of stay, days (median, range)	6 (1–45)	5 (1–56)	5 (1–91)	.02
ICU admission** (n,%)	77/277 (27.8%)	20/48 (41.7%)	198/655 (30.2%)	.03
Oxygen use (n,%)	Mechanical ventilation: 41 (14.3%) BPAP: 2 (0.7%) Supplemental: 173 (60.5%) None: 70 (24.5%)	Mechanical ventilation: 11 (20.4%) BPAP: 0 Supplemental: 29 (53.7%) None: 14 (25.9%)	Mechanical ventilation: 91 (13%) BPAP: 4 (0.6%) Supplemental: 435 (62.1%) None: 170 (24.3%)	.79
Diabetes (n,%)	156 (54.5%)	31 (57.4%)	311 (44.4%)	.006
COPD* (n,%)	98 (34.3%)	15 (27.8%)	114 (16.3%)	< .0001
Cardiovascular disease*** (n,%)	177 (61.9%)	39 (72.2%)	232 (33.1%)	< .0001
Kidney disease (n,%)	136 (47.6%)	25 (46.3%)	190 (27.1%)	< .0001
Cancer (n,%)	74 (25.9%)	12 (22.2%)	91 (13%)	< .0001
Obesity**** (n,%)	166 (58%)	26 (48.1%)	311 (44.4%)	< .0001
Organ Transplant (n,%)	6 (2.1%)	2 (3.7%)	8 (1.1%)	.23
Sickle cell anemia (n,%)	1 (0.3%)	0	3 (0.4%)	N/A

*PPI Proton pump inhibitor, *COPD* Chronic obstructive pulmonary disease.

**Denominators are reduced due to “N/A” responses for certain patients.

***Cardiovascular disease is a composite of cardiomyopathy, congestive heart failure, and coronary artery disease.

****Defined as BMI > 30 kg/m2.

*****Based on separate chi square or Kruskal Wallis tests, as appropriate; “N/A” indicates insufficient sample sizes for statistical compari.

**Table 5:** Active PPI use: outcomes based on PPI dosage (n = 286)*.

	Low (n = 70)	Standard (n = 190)	High (n = 26)	*p*-value**
Hospital admission within two weeks of COVID testing (n,%)	68 (97.1%)	182 (95.8%)	25 (96.2%)	.88
ICU admission (n,%)	16 (22.9%)	52 (27.4%)	9 (34.6%)	.50
Hospital length of stay, days (median, range)	1 (1–33)	3 (1–32)	3.5 (1–45)	.07
Oxygen use (n,%)	Mechanical ventilation: 7 (10%) BPAP: 1 (1.4%) Supplemental: 47 (67.1%) None: 15 (21.4%)	Mechanical ventilation: 28 (14.7%) BPAP: 1 (0.5%) Supplemental: 106 (55.8%) None: 55 (28.9%)	Mechanical ventilation: 6 (23.1%) BPAP: 0 Supplemental: 14 (53.8%) None: 6 (23.1%)	.47
30-day mortality (n,%)	10 (14.3%)	37 (19.5%)	7 (26.9%)	.35

*PPI Proton pump inhibitor.

**Based on separate chi square or Kruskal Wallis tests, as appropriate.

Additionally, data was also collected on the use of histamine receptor antagonist (H2RA) within the study’s sample. For active and past PPI users taking H2RA medications (n = 206), famotidine was most frequently consumed (170, 82.5%), followed by ranitidine (34, 16.5%); and cimetidine (2, 1.0%). 55 patients were found to be taking H2RAs, but not PPIs. Unfortunately, due to this limited subgroup sample, analysis was unable to be performed to yield statistically significant results.

## Discussion

Many hypotheses have been developed theorizing the potential effect of PPI therapy on severity of COVID-19 infection based on prior data examining the use of PPIs and their associations with infection risk^15^. Previous studies such as, Moayyedi et al., have shown that PPI therapy is associated with increased risk of enteric infections, possibly secondary to suppression of gastric acid secretion^16–19^. Additional studies have also proven inactivation of viruses in extreme acidic or basic environments and stabilization in a neutral environment often created by PPI and other forms of antacid medications^20,21^. SAR-CoV2 has been showed to invade the body through the gastrointestinal tract via the angiotensin converting enzyme-2 (ACE-2) receptor. In addition to being found on cells in the epithelium of the lungs, ACE-2 is also abundantly found on the enterocytes in the gastrointestinal tract providing them with a point of invasion^22,23^. Therefore, these findings have prompted theoretical concern that use off PPI therapy can place individuals not only at increased risk of COVID-19 infection but also of severe disease.

Our large, single network retrospective study examined the relationship between PPI therapy and the severity of COVID-19 infection. After adjusting for relevant patient demographics and clinical variables, neither active nor past PPI use significantly predicted hospital admission within two weeks of COVID testing or 30-day mortality. For patients admitted to the hospital, active PPI users had slightly higher median length of stay, and but both active and past PPI users had a greater frequency of comorbid conditions compared to non-users. Additionally, dosage of PPI therapy (low, standard, and high) was compared but was not found to be associated with more frequent hospital admissions within two weeks of COVID testing, requirement for ICU admission, or increased length of hospital stay.

Recent literature has found conflicting results regarding the association between PPIs and COVID-19 outcomes. One of the first and largest studies evaluating the association was the Korean Nationwide Cohort Study completed by Lee et al. which involved a sample size of 234,427 patients^24^. Overall, this study concluded that PPI use may not increase susceptibility to SARSCo-2 infection but placed individuals at increased risk of severe COVID-19 infection. Results of our studied differed in that there was no positive correlation between PPI use and COVID-19 severity, but rather significant correlations were seen with various comorbidities through completion of regression modeling. Although the Korean Cohort Study did make note of their sample’s baseline characteristics and comorbidities and their strength lies in the study’s large sample size, the study lacked insight into the potential confounding variables that they left unmeasured. In addition to completing a multi regression analysis to determine the independent effect of PPI usage, our study further aimed to look at the effect of age and comorbid conditions, including obesity or increased BMI, which are confirmed risk factors for COVID-19^6,7,25^. Through bivariate analyses, results did show that age, diabetes, COPD, cardiovascular disease, obesity, and kidney disease were independently and significantly associated with increased admission while age, diabetes, and COPD were associated with increased mortality. Although no significant association was seen between COVID and obesity, this variable was highlighted considering it is a well-established risk factor for viral infections^25,26^. Given these results, one must question whether the Korean Cohort Study’s results and overall conclusions would have differed if their analysis paralleled ours.

The publication of this large preliminary study stimulated further research and publication of subsequent studies investigating the impact of PPI therapy on COVID-19 infection^27–30^. Studies completed by Ramachandran et al., Zhou et al. and, Luxenburger et al. all exclusively looked at the use of gastric acid suppressants in the era of COVID-19 and associated PPI use to severe COVID-19 infection^28–30^. The retrospective cohort study completed by Ramachandran et al. is an alley United States study completed in Brooklyn, New York that examined pre-hospitalization PPI use early during the pandemic^28^. Similar data in comparison to our study was collected but the study included a much smaller sample of 295 hospitalized patients limiting the study’s impact. The study did associate PPI use with higher risk of mortality but solely looked at hospitalized patients and thus was unable to comment on the association between PPI usage and need for hospitalization. A very similar study completed by Luxenberger et al. also included a smaller sample of 152 patients that focused largely on the fact that PPI use was a significant risk factor for development of secondary infections in COVID-19 patients^29^. However prior to COVID-19, studies have previously shown that PPI use as well as gastrointestinal reflux disease (GERD) itself increases individuals risk of enteric infection in general making this finding trivial as it is not specific to COVID-19^12,29^. Lastly, Zhou et al., conducted a territory wide study involving 4445 patients to investigate PPI use as well as Famotidine use in COVID in Hong Kong public hospitals^30^. This was a larger study, conducted much like our own, but unfortunately completed in Hong Kong targeting a different patient demographic and necessitating additional studies to be completed on Americans.

Although large scale studies regarding PPI use and COVID-19 have been completed outside of the United States, the largest American study completed to date is a self-administered online survey study by Almario, CV et al. targeting patients with a history of gastrointestinal symptoms and involving 53,130 adult participants^31^. Completion of regression analysis revealed that those who reported daily PPI use were found to have significantly increased odds of reporting a positive test result. However, the validity of study can be argued given the inherit nature of a survey study. Moreover, there is even greater concern that it may not appropriately represent the American population^32^. 74% of the patients who tested positive for COVID-19 in the study sample were aged 30–39 years old^31^. Currently out of the 80 million cases in the United States, only 17% of those total cases have occurred in patients in the 30–39 age group^1,31^. Therefore, this study’s patient majority does not parallel that of the United States. This could largely be because this study was conducted by an internet survey, unintentionally targeting a younger patient demographic. However, it is also likely because they failed to consider those who died from COVID-19, a key patient group that disproportionally includes patients 65 years and older^33^. Our study had an overall mean age of 52.6 years and appropriately considered those patients with a 30-day mortality. Additionally, our study also helps to provide information regarding hospitalization and clinical outcomes of patients who did test positive for COVID-19 and is the largest United States retrospective cohort study to do so. Therefore, our study helps to provide a better representation of the American population by eliminating subject bias and augmenting prior studies results with clinical information.

We recognize that this study has limitations in addition to its strengths. Given the retrospective nature of our analysis, our data gathering relied heavily on proper patient charting and documentation of comorbid conditions and PPI usage and dosing. We were unfortunately unable to analyze and include clinical features which persisted beyond hospitalization. Moreover, we were unable to control for certain comorbidities in our regression model such as organ transplantation and sickle cell anemia which may have reduced clinical robustness. Lastly, our data collection was limited to our single hospital network’s patient data and therefore must be validated across multiple sites to ensure external generalizability. Our findings are associations, and causality cannot be determined given the nature of the study design. However, the strength of our study lies in the fact that it is the largest retrospective cohort study to be completed on PPI’s effects on COVID19 infection in the United States to date. This study was completed within our large multi-hospital network in Eastern Pennsylvania and our sample includes a large catchment area that covers urban, suburban and rural communities, with a stable patient population. We were able to collect more granular data than prior studies given the ability to chart review and utilize electronic medical records.

## Conclusion

Use of PPI therapy has been shown to increase individuals risk of infection and has raised theoretical concern that it can lead to severe COVID-19 infection. Our data suggests that PPI therapy is not associated with an increased risk of severe COVID-19 infection including need for hospitalization or overall, 30-day mortality. Although more data is necessary in order to include PPI use in risk stratification models for COVID-19, clinicians should recognize that based off our data that COVID-19 should not change their clinical management and prescription of PPI therapy.

## Data Availability

The datasets used and/or analyzed during the current study are available from the corresponding author on reasonable request. 16.
